# Sparsity Adaptive Matching Pursuit Detection Algorithm Based on Compressed Sensing for Radar Signals

**DOI:** 10.3390/s17051120

**Published:** 2017-05-13

**Authors:** Yanbo Wei, Zhizhong Lu, Gannan Yuan, Zhao Fang, Yu Huang

**Affiliations:** 1 College of Automation, Harbin Engineering University, No. 145 Nantong Street, Harbin 150001, China; weiyanbo@hrbeu.edu.cn (Y.W.); yuangannan@hrbeu.edu.cn (G.Y.); fangzhao@hrbeu.edu.cn (Z.F.); 2 College of Science, Harbin Engineering University, No. 145 Nantong Street, Harbin 150001, China; huangyu@hrbeu.edu.cn

**Keywords:** compressed sensing, radar signal, sparsity adaptive, target detection

## Abstract

In this paper, the application of the emerging compressed sensing (CS) theory and the geometric characteristics of the targets in radar images are investigated. Currently, the signal detection algorithms based on the CS theory require knowing the prior knowledge of the sparsity of target signals. However, in practice, it is often impossible to know the sparsity in advance. To solve this problem, a novel sparsity adaptive matching pursuit (SAMP) detection algorithm is proposed. This algorithm executes the detection task by updating the support set and gradually increasing the sparsity to approximate the original signal. To verify the effectiveness of the proposed algorithm, the data collected in 2010 at Pingtan, which located on the coast of the East China Sea, were applied. Experiment results illustrate that the proposed method adaptively completes the detection task without knowing the signal sparsity, and the similar detection performance is close to the matching pursuit (MP) and orthogonal matching pursuit (OMP) detection algorithms.

## 1. Introduction

In recent years, in order to improve the image resolution, radar systems generally use a larger signal bandwidth. Under the limitation of Nyquist sampling theorem, the system needs to sample the received data at a high rate, which results in a large amount of data [1,2]. This problem not only imposes a higher requirement on A/D converters but also brings difficulties to data storage and transmission.

Compressed Sensing (CS) theory overcomes the inherent defects of traditional radar and solves the problems of the high sampling rate, large data amount and real-time processing difficulties faced by traditional high-resolution radar [3,4]. The prior condition for applying CS theory is that the signal itself is sparse or in a transformation can be sparsely represented [5]. Based on the emerging CS theory, the original signal could be restored almost perfectly under the low sampling law by using the prior knowledge of the sparsity of the original signal. Therefore, both the amount of data to a certain extent and the requirements for sampling equipment are reduced when dealing with the sparse problem based on the CS theory.

So far, the CS is mainly focused on signal recovery and reconstruction [6,7,8,9]. The radar imaging algorithm based on the CS theory is mainly through randomly sampling the echo data required for imaging and then reconstructing the radar image with the sampled value of random sampling [10,11].

Although CS is closely focused on signal recovery and reconstruction, many signal processing problems such as detection, classification and estimation do not require a complete recovery signal. These problems could be solved by extracting statistical information directly from a small number of random projections without reconstructing the signal [12].

For the target detection of a radar signal, the detection problem will become complicated and cause an unnecessary waste of resources if the target detection is performed after the original signal is reconstructed. The signal detection task could be implemented by directly using the sampled value of CS, since the structure and information of the original signal are well maintained in the sample value [3,13,14,15]. Then, the sampled value can be directly used for signal detection and estimation by the strictly theoretical derivation proved in [16]. The partial theoretical derivation is also presented in [17], and the target detection based on the position information of the non-zero elements of the sparse signal without reconstructing the whole signal is proposed in [17].

Based on the characteristics of the acquired marine radar images with high resolution and a large amount of data, the experiment of target detection in the X-band navigation radar image is studied in this paper. The small ships, buoys, sea surface protruding rocks and other small objects which stands for a bright, continuous regional or isolated higher echo area can often be encountered in the acquisition process of the original radar image. The target of a ship in the radar echo image denotes isolated bright spots. Since the targets in the radar echo images are tiny in the whole scene and the number of targets is small, the electromagnetic properties of targets are similar to the sparse character of the CS theory. The electromagnetic characteristic of the targets can be represented by a few important scattering centers, which is consistent with the requirement of sparsity in CS theory and makes the scattering coefficient vector sparse [3]. Thus, it is possible to apply the CS theory to the radar imaging.

Currently, the research results based on the CS on the radar signal detection are relatively rare. In [12], the CS theory is introduced into the analog signal detection for the first time, and a detection algorithm based on matching pursuit (MP) is proposed. During each iteration, the closest selection is selected from the atomic library. The residual atoms are used as the supporting atoms. Subsequently, the MP signal detection algorithm in the noise case is investigated. In [18], the CS theory was firstly applied to the detection and estimation of the radar signal, and then the MP detection algorithm was proposed to solve the problem of target detection of the radar signal.

According to the shortcomings of the MP method that the projection on the set of selected atoms is non-orthogonal and the estimated characteristic value has a big fluctuation [19], the orthogonal matching pursuit (OMP) detection algorithm based on the sampled value was proposed to reduce the number of iterations. Simultaneously, the simulated radar data was used to verify the detection performance [19]. Based on the prior information, the location information of the interesting signal is acquired and the sparse relativity of location information is used as the criteria to complete detection task [20]. However, the detection threshold depends on the number of iterations.

Both MP and OMP detection algorithms require the sparsity of signal as the prior knowledge to determine the number of iterations. However, in practice, it is often impossible to know the sparsity of the signal in advance. Our primary goal in this paper is to propose a novel sparsity adaptive matching pursuit (SAMP) detection algorithm to efficiently and effectively execute the detection task by updating the support set and gradually increasing the sparsity to approximate the signal without knowing the prior knowledge of the signal sparsity. This paper is organized as follows: Section 2 presents the signal detection theory based on CS. The OMP detection algorithm based on CS is shown in Section 3. The detailed novel sparsity adaptive radar signal detection algorithm based on the CS is given in Section 4. In Section 5, the validity of the proposed method is investigated by the acquired radar data. Finally, the discussion and conclusion are summarized in Section 6 and Section 7, respectively.

**Notation**: Scalars, vectors and matrices are expressed by regular, bold lowercase letters and bold uppercase letters, respectively. $\left|\right|\cdot {\left|\right|}_{\infty }$ indicates the infinite norm. C denotes the complex domain. 〈*〉 denotes the inner product.

## 2. The Signal Detection Theory Based on CS

### 2.1. The CS Theory

Suppose that the signal $x\in R^{N}$ or $C^{N}$ is K-sparse, the signal x can be expressed as

(1) $$x_{N\times 1}=\Psi_{N\times N}\theta_{N\times 1},$$

where K≪N, Ψ is the sparse dictionary and $\theta_{N\times 1}$ is the coefficient vector.

In the CS theory, the sparse signal x is projected onto the low-dimensional sensing matrix by non-correlation measurement. Then, the sampled value y can be described as

(2) $$y_{M\times 1}=\Phi_{M\times N}x_{N\times 1}=\Phi_{M\times N}\Psi_{N\times N}\theta_{N\times 1},$$

where M≪N and the sensing matrix Φ satisfies the Restricted Isometry Property (RIP) condition [17]. Suppose a constant δ∈(0,1) exists, for the signal x, if ${\left||x|\right|}_{0}<K$, thus the RIP condition can be shown as

(3) $${\left(1-\delta \right)||x||}_{2}^{2}\leq {\left||\Phi x|\right|}_{2}^{2}\leq {\left(1+\delta \right)||x||}_{2}^{2}.$$

Given V=ΦΨ , then Equation (2) can be simplified as

(4) $$y_{M\times 1}=V_{M\times N}\theta_{N\times 1}.$$

Here, it is observed clearly that the original signal structure information is maintained in the sampled value y. Therefore, the detection task could be realized by processing the sampled value. In general, a solution with a sparse structure is achieved by solving the norm optimization problem from Equation (4). Since finding a sparse solution of Equation (4) is an ill-posed problem, a solution with the sparse structure is found by solving an approximate $l_{1}$ norm optimization problem that is more robust to the error and noise. The $l_{1}$ norm optimization problem is given by [3,21]

(5) $${\min||\theta ||}_{l_{1}}\;s.t.\;y=V\theta .$$

### 2.2. The Detection Principle Based on CS

In the conventional target detection methods, the detection task is carried out by distinguishing the following two hypotheses:

(6) $$H_{0}:x=n\;\mathrm{vs}.\;H_{1}:x=s+n,$$

where $H_{0}$ denotes the case that only has noise without a signal, and $H_{1}$ denotes the case of including noise and signals. In this paper, $s\in R^{N}$ is the known target signal of the marine radar image, $n\in R^{N}$ is the system thermal noise and sea clutter noise of the image. Although the noise is not sparse in the transform domain Φ, the target signal is sparse. Thus, the Equation (6) can be rewritten as

(7) $$H_{0}:x=n\;\mathrm{vs}.\;H_{1}:x=\Psi \theta +n.$$

Then, the two hypotheses can be distinguished by measuring the below coefficient vector θ

(8) $$H_{0}:\theta =0\;\mathrm{vs}.\;H_{1}:\theta \neq 0.$$

Supposing that Equation (8) can be used to realize the feature judgment, the two hypotheses in Equation (6) are distinguished [12,19]. In practice, due to noise and system error, θ cannot be zero vector even in the absence of the target signal. Therefore, a threshold is required to distinguish the two hypotheses between the presence and absence of the target signal. Then, we have

(9) $$H_{0}:||\hat{\theta }{\left|\right|}_{\infty }\leq \gamma \;\mathrm{vs}.\;H_{1}:||\hat{\theta }{\left|\right|}_{\infty }>\gamma ,$$

where γ is the threshold.

## 3. The OMP Detection Algorithm Based on CS

The greedy class iterative detection algorithm is widely used because of its simple structure and small amount of computation, taking into account the operating efficiency and sampling efficiency. In the case where there is a target in the signal, the characteristic value estimated of the sample value based on the greedy MP iterative detection algorithm has a big fluctuation, which affects the detection performance [19,22].

Against the shortcoming of MP detection algorithm, the OMP detection algorithm is proposed in [19,20]. In each iteration process, the selected atoms are orthogonally processed, which ensures the optimality of each projection, reduces the number of iterations and updates the sparse coefficients in the process of projection. The OMP detection algorithm can get the optimal projection of the sampled value on the selected column and accelerate the convergence rate compared to the MP detection method. The OMP algorithm is as follows: assume the residual $r_{0}=y$, matrix $V_{0}=\emptyset$, the initially iterative number t=1, and then

1. Select the column from the matrix V that has the largest correlation with the residual:
  (10) $$n_{t}=\max_{i=1,2,\cdots ,N}\left(\right|\langle r_{t-1},v_{i}\rangle \left|\right),$$
  where $n_{t}$ is the selected column from matrix V during the iteration, $n_{t}\in \left\{1,2,\cdots ,N\right\}$, and $v_{i}$ represents the *i*-th column of matrix V.
2. Update the selected column set
  (11) $$V_{t}=V_{t-1}\cup V_{n_{t}}.$$
3. By solving the least squares problem, the sparse coefficient estimated $\hat{\theta }$ is updated by
  (12) $$\hat{\theta }=\arg\min_{\theta }||y-V_{t}\theta {\left|\right|}_{2}^{2}.$$
4. Update residual $r=y-V_{t}\hat{\theta }.$
5. Update the iteration times t=t+1. if t<T, go to step 1 to continue the iteration; otherwise, go to step 6.
6. If $\left|\right|\hat{\theta }\left|\right|>\gamma$, select $H_{1}$; otherwise, select $H_{0}$.

The algorithm demands defining the number of iterations that is determined by the sparsity of the targets. In the case where the signal sparsity is unknown, the number of iterations can not be estimated in advance. If the number of iterations is too large, it will increase the amount of computation, waste time and then reduce the efficiency of operation. Otherwise, the sparse coefficients can not be recovered well, which may lead to reducing the signal detection success rate. Simultaneously, whether or not the signal contains targets, the OMP and MP detection algorithms must be iterated *T* times to obtain the sparse coefficients, and then they are compared with the threshold. For a signal containing only noise, it may just need one iteration to make the judgment.

In practice, the signal sparsity is often not known in advance. For example, we can not know in advance the number of fishing boats in the acquired radar images, since they may enter or leave the observation area of the radar at any time. Therefore, it will present an important practice value if the task of signal detection is completed in the case that the signal sparsity is unknown.

## 4. Sparsity Adaptive Radar Signal Detection Algorithm

For the MP and OMP detection methods, it is demanded to design suitable iteration stopping conditions based on the sparsity *K* for achieving better detection performance [18,19].

Aiming at the shortcomings of the existing detection algorithms, based on the SAMP reconstruction algorithm [23], we propose a SAMP target detection algorithm which is not a straightforward reconstruction algorithm and is described as follows: assuming the residual $r_{0}=y$, the list of candidate support set $c_{0}=\emptyset$, $V_{0}$ is an empty set, *S* is the iterative step size, the initially iterative number t=1, the number of stage j=1 and the size of support set I=j*S, then

1. The selected *I* columns from matrix V, which have the highest correlation with the residual, are determined by
  (13) $$n_{t}=\max_{i=1,2,\cdots ,N}\left(\right|\langle r_{t-1},v_{i}\rangle \left|,I\right),$$
  where $n_{t}$ stands for the index set of the selected *I* column, $n_{t}\subset \left\{1,2,\cdots ,N\right\}$, and $v_{i}$ represents the *i*-th column of matrix V.
2. Construct the list of candidate support set
  (14) $$c_{t}=c_{t-1}\cup n_{t}.$$
3. Select *I* columns from $V_{c_{t}}$ that have the highest correlation with the residual and construct the new support set $V_{t}=V_{d_{t}}$
  (15) $$d_{t}=\max_{t=1,2,\cdots ,N}\left(\right|\langle r_{t-1},V_{c_{t}}\rangle \left|,I\right).$$
4. By solving the least squares problem, the sparse coefficient estimated $\hat{\theta }$ is updated by
  (16) $$\hat{\theta }=\arg\min_{\theta }||y-V_{t}\theta {\left|\right|}_{2}^{2}.$$
5. Update residual $r=y-V_{t}\hat{\theta }.$
6. Judge whether or not the iteration stopping condition is satisfied based on the energy difference of sparse coefficient. Step 7 is executed if the stop condition is not satisfied; otherwise, quit the iteration and go to step 8.
7. Determine whether $\left|\right|r_{t-1}{\left|\right|}_{2}\leq \left|\right|r_{t}{\left|\right|}_{2}$ is true. If it is true, update the iteration number of stage j=j+1 and the support set size I=j*S, and then go to step 1 to continue the iteration. Otherwise, update the support set $V_{c_{t-1}}=V_{t}$, residual $r_{t}=r$, and the iteration times t=t+1, and then go to step 1 to continue the iteration.
8. If $\left|\right|\hat{\theta }\left|\right|>\gamma$, select $H_{1}$; otherwise, select $H_{0}$.

In the above SAMP detection algorithm, it is critical to design a reasonable iterative stopping condition that is different to the reconstruction method. In the research of radar image reconstruction, we usually compare the energy difference of the reconstructed signal of two successive phases with the threshold as the iteration stopping criteria. Inspired by this, for the task of target detection, the stopping condition here is designed according to the difference of the sparsity coefficient of two successive phases $\left|\right|{\hat{\theta }}_{t}-{\hat{\theta }}_{t-1}\left|\right|$.

Compared to the current MP and OMP algorithm, the most prominent feature of the SAMP detection algorithm is that the SAMP detection algorithm, which estimates the sparsity through a variable step size and gradually increases the support set to approximate the original signal, could automatically complete the detection task without inputting the number of iterations.

In each iteration, the SAMP detection algorithm firstly selects some atoms from the measurement matrix which perfectly match the residuals $r_{t}$ of the original signal, secondly forms a new candidate support set by combining the newly selected atoms with the candidate support set obtained in the last iteration, and then updates of the support set by choosing the atoms that match the residue. Meanwhile, the novel detection algorithm introduces the idea of variable step size. By setting the step size, it is possible to select more than one atom once. Thus, it has higher operating efficiency compared to MP and OMP detection methods.

In the SAMP detection algorithm, since *K* is unknown, it is also critical to choose a reasonable step size *S*. Smaller step size is a safe choice, but the running speed of the algorithm detection rate will be greatly reduced due to it consuming a lot of time. If *S* is too large, the size of the support set is likely to exceed the sparsity, and then results in the decrease of the detection success rate.

## 5. Experimental Results and Analysis

### 5.1. The Experimental Data

To examine the validity of the SAMP detection algorithm, the data collected in 2010 at Pingtan, which is located on the coast of the East China Sea, were applied for validation and analysis [24,25,26]. In our experiment, the software of MATLAB (R2010b), which runs on the Microsoft Windows Xp Professional operating system is developed to implement the MP, OMP and our SAMP detection algorithms. The processor of the computer is 2 GHz Inter(R) Core(TM) CPU i5-3337U and the installed RAM is 8 GB.

**Figure 1:**
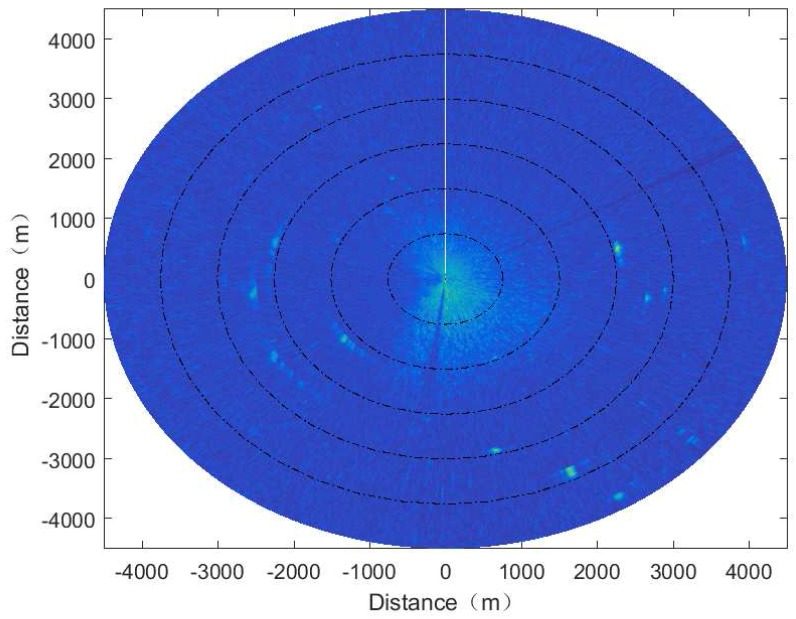
The acquired X-band marine radar image.

The original image acquired using the X-band marine radar, which contains the echoes of the target signal in the distance and azimuth direction, is shown in Figure 1. The echo intensity in the center of the image is the sea clutter, and the isolated bright spots are the echo of vessels. In order to better detect the targets from the radar signal, the co-frequency interference in the radar images here has been suppressed. The parameters of the X-band marine radar are shown in Table 1.

**Figure 2:**
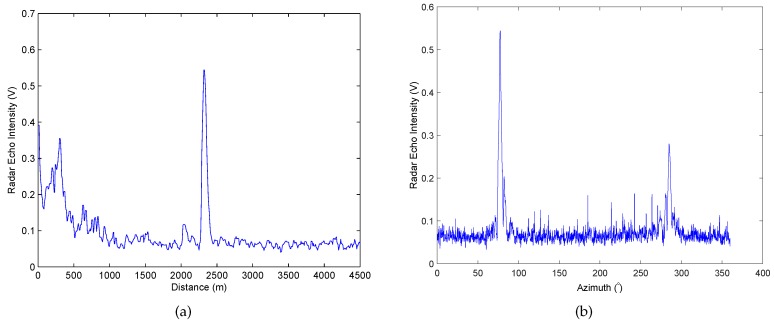
The radar echo intensity in respective distant and angular direction. (**a**) The radar echo intensity of 300-th line in distance direction; (**b**) The radar echo intensity at 2400 m in angular direction.

Figure 2 shows the signal characteristics of the target in the distance and azimuth in the marine radar image, which is sparse compared to the whole signal. From Figure 2, it can be observed that the signal characteristics of the small targets appearing on the sea clutter image are as follows.

In the distance direction, i.e., the radial of the radar image, the echo intensity of the fishing vessel location stands for several consecutive strong points. The 300-th signal line in the radar image that contains the target of the fishing vessel is presented in Figure 2a. It can be observed clearly that the vessel target exists at the position of approximate 2400 m. In the azimuth, i.e., the angular direction of the sea radar image, the echo intensity of the fishing vessel location also appears as several consecutive strong points in Figure 2b.

Both from Figure 1 and Figure 2, it can be observed that the targets such as the fishing boat in the navigation radar image denote the isolated large bright spots, which is smaller compared with the whole scene, and the number of bright spots is very small. The electromagnetic properties of the targets in the radar signal are in good agreement with the sparseness requirements of the CS, which provides the possibility of the application of the CS theory in radar imaging.

In the experiment, Ψ is a transform matrix, and Φ is a sparse circulant matrix with independent and identical distribution [27,28]. Due to the randomness of the compression matrix, each set of data is repeated 1000 times. The data in distance direction as the input signal is applied to the target detection experiment. The vessels that enter and leave the port denote the targets of the radar images in our experiment.

### 5.2. Experimental Results

**Figure 3:**
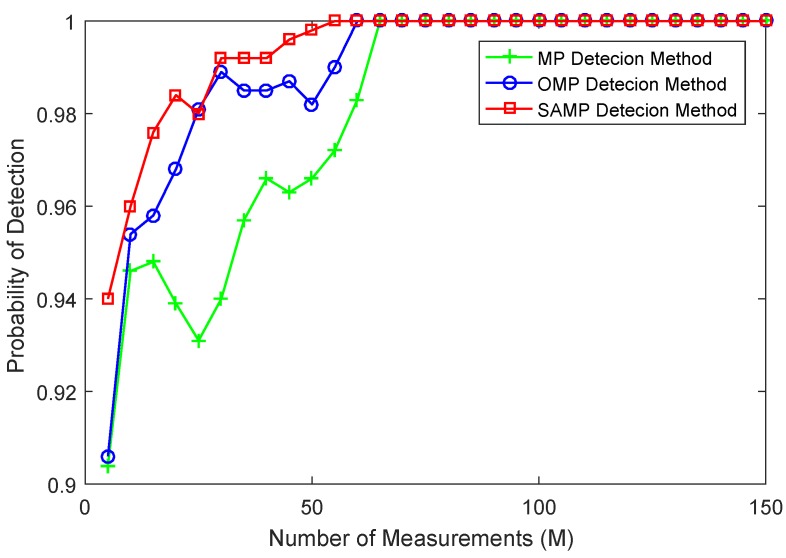
The comparison of the detection success rate versus sample points for different detection algorithms.

In the experiment, N=512 is the length of the interesting signal. Here, the comparison of the detection success rate versus sample points for different detection algorithms is studied. Under the same threshold, the performance of the detection algorithms is shown in Figure 3, where the signal-to-noise ratio (SNR) is SNR=15, the number of iterations of MP and OMP methods is T=10, the step size of SAMP method is S=3 and the threshold γ=0.3 is obtained by the optimal detection based on Monte Carlo simulations. From Figure 3, it can be seen that all the MP, OMP and SAMP detection algorithms show the same success rate when the number of measurements *M* is greater than 60. However, the SAMP detection algorithm in the same compression rate (M/N) presents a significantly higher success rate compared to MP and OMP detection methods when *M* is less than 60. When *M* is less than 10, all of these detection algorithms show a lower detection success rate, since the sample value contains less information of the original signal. In addition, the detection success rate of MP and OMP methods has a big fluctuation. Since the SAMP method can be viewed as a framework for MP and OMP and can update the support set by removing the atoms selected in the iteration, the SAMP method presents better performance than that of MP and OMP detection methods. Figure 3 also certifies that these algorithms could complete the detection task even with significantly fewer measurements.

**Figure 4:**
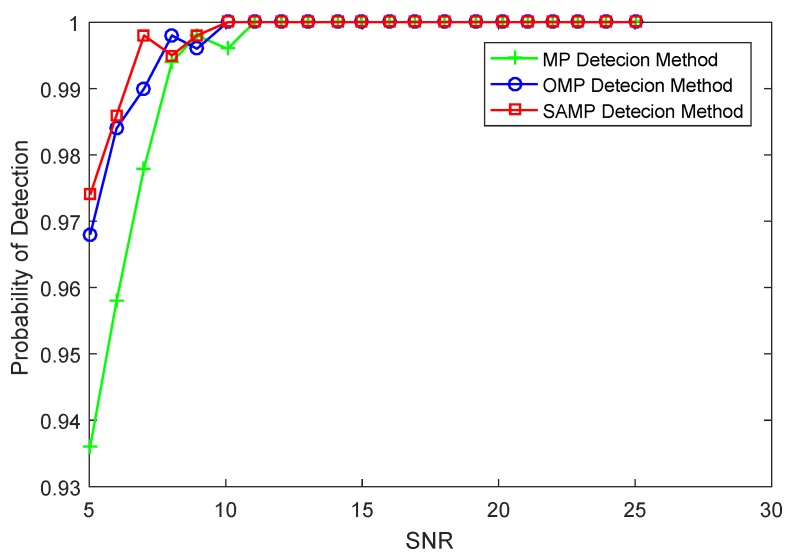
The comparison of success rate versus SNR.

Here, we discuss the relation between the detection success rate and SNR under the same measurements and threshold. The dimension of sampled value is M=102, the detection threshold is γ=0.3, the number of iterations of MP and OMP methods is still T=10, and the step size of SAMP method is S=3. It should be mentioned that the SNR of a signal is adjusted by modifying the magnitude of the target in the experiment since the radar echo intensity of target is fixed. Under the same threshold, the performance of the detection algorithms is shown in Figure 4. It can be seen that all of the MP, OMP and SAMP detection algorithms show the same success rate when the SNR is greater than 12. It is observed clearly that the SAMP detection algorithm has better anti-noise ability. The SAMP detection algorithm presents a slightly higher detection success rate compared to the OMP detection method when SNR is less than 12. The OMP detection algorithm can get the optimal projection of the sampled value on the selected column. In addition, the SAMP can be seen as a generalized OMP algorithm. In addition, the SAMP detection algorithm introduces the idea of atomic backtracking and selects the atoms from the candidate support set in each iteration. Moreover, the SAMP detection algorithm presents a significantly higher detection success rate compared to the MP detection method when SNR is less than 12. The MP detection method has worse detection performance, since the projection on the set of selected atoms is non-orthogonal. When SNR is less than 5, all of these detection algorithms show a lower detection success rate. Thus, to complete the detection task is our further research under low SNR.

**Figure 5:**
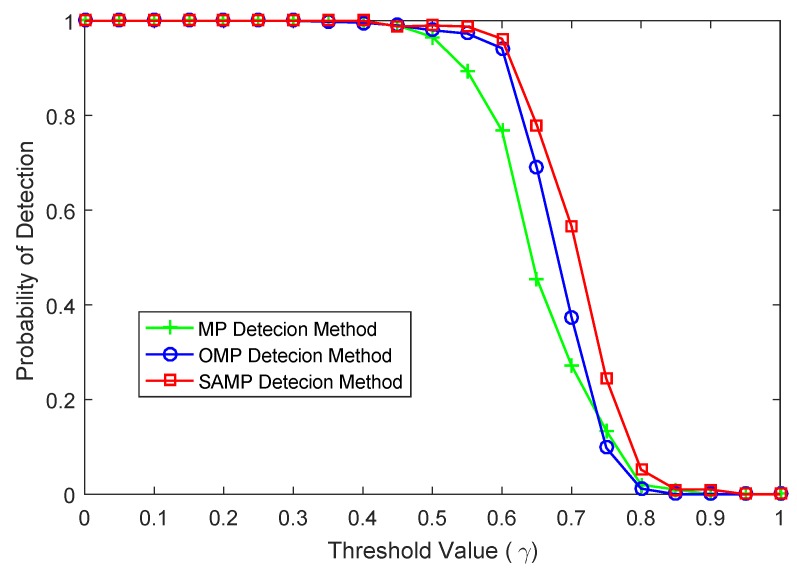
The comparison of success rate versus threshold.

The detection success rate versus threshold under the same sampled value and SNR is shown in Figure 5. Here, M=102, SNR=15, the number of iterations of MP and OMP methods is still T=10, and the step size of SAMP method is S=3. When the threshold is relatively small and only noise presents, it is easy to consider noise as a signal for the detection algorithms. From Figure 5, it can be seen that the performance of the SAMP detection algorithm is also higher than that of MP and OMP detection methods in the effective threshold interval. All of the MP, OMP and SAMP detection algorithms show the same success rate when the threshold value γ is less than 0.4. However, both of the OMP and SAMP detection algorithms present a significantly higher success rate compared to MP detection methods when γ∈(0.5,0.65). In this threshold interval, the SAMP detection method almost has the same success rate with that of OMP detection method. The reason may be that the selected atoms of SAMP and the selected atom of OMP among each iteration are orthogonal. When the threshold is greater than 0.65, all of these detection algorithms show a lower detection success rate, since the threshold is unreasonable and the target signal is regarded as noise.

**Figure 6:**
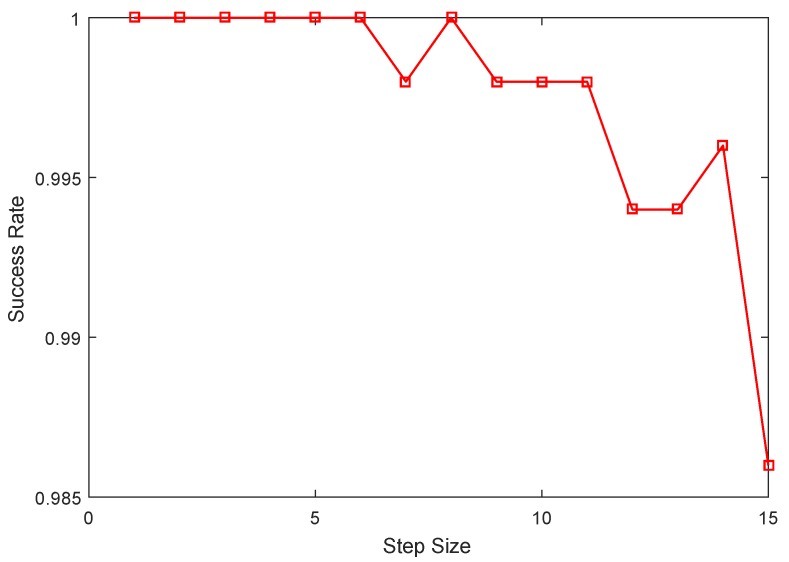
The comparison of success rate versus step size.

Although the SAMP detection method presents a lower success rate compared to MP and OMP detection algorithms, the SAMP detection algorithm could complete adaptively the detection task and illuminates the approximate detection performance. For the SAMP method, the detection success rate versus the iteration step size that is presented in Figure 6 is investigated under the condition that the dimension of sampled value M=102, threshold γ=0.3 and SNR=15. Figure 6 shows that the detection success rate of the SAMP detection algorithm begins to decrease gradually when step size S>6. The experimental results also hint that the selection of iterative steps heavily depend on the sparsity of the signal.

**Figure 7:**
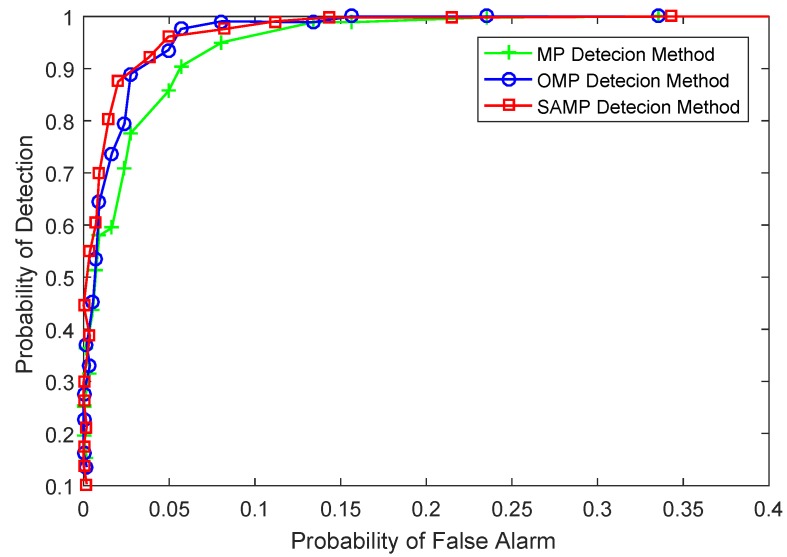
The performance of different detection algorithms.

The receiver operating characteristic (ROC) curve is illustrated in Figure 7. It is observed clearly that the SAMP detection algorithm presents a significantly higher detection success rate compared to the OMP detection algorithm. The performance of the SAMP detection algorithm is close to that of the OMP detection method when the probability of false alarm $P_{f}\in \left(0.05,0.15\right)$, but the SAMP detection algorithm has better detection performance than the MP detection algorithm when $P_{f}$ is less than 0.05. The SAMP method can be viewed as a framework for MP and OMP and chooses the optimal projection of the sampled value on the selected column in each iteration. In addition, the SAMP detection algorithm introduces the idea of atomic backtracking. From Section 4, it can be observed clearly that the SAMP detection algorithm selects atoms from the candidate support set. Therefore, the SAMP detection algorithm illuminates better performance than the MP and OMP detection algorithms. The experimental results based on the real marine radar data containing sea surface targets present that the proposed algorithm not only adapts to realize the target detection task but also presents satisfactory detection performance.

In the simulation, the performance of different detection methods using real radar data is researched. Meanwhile, the validity of the SAMP detection algorithm, which can realize the signal detection without the prior knowledge of signal sparsity compared to MP and OMP detection algorithms, is certified.

The computing time performance of MP, OMP and SAMP detection algorithms is shown in Table 2, where the probability of detection and the average runtime of the detection methods are denoted as $P_{d}$ and *T*, respectively. The step size of the SAMP method in Table 2 is *S* = 3. From Table 2, it is observed clearly that the SAMP detection algorithm consumes slightly shorter computation time compared to MP and OMP detection methods, since the SAMP detection algorithm could select multiple atoms in the process of updating the support set. The computing time performance of the SAMP detection algorithm with respect to various step size is investigated in Table 3. From Table 3, it is observed clearly that the SAMP detection algorithm with small step size consumes slightly longer runtime compared to that with large step size, since a larger number of iterations is required for small step size.

## 6. Discussion

Both the MP and OMP detection algorithms require inputting the number of iterations based on the sparsity of the signal. In addition, the effectiveness of these methods is validated by using the simulated radar data. The prominent advantage of the SAMP algorithm proposed in this paper is that it does not require inputting the sparse degree as the initial reference of the number of iterations, and expands the support set by accumulating at each stage of iteration. Moreover, in the process of iteration, the detection task is completed by measuring a close degree to the original signal sparsity coefficient.

Based on theoretical analysis and experimental results, the SAMP detection algorithm achieves better detection performance than MP and OMP algorithms. It certifies that the designed iterative stopping condition based on the energy difference of reconstructing the sparse coefficient is reasonable for radar signal. In addition, more experimental tests are still needed to further validate the effectiveness of the SAMP algorithm for radar signal detection.

For the noise signal in this paper, although it is necessary to iterate the fixed number of iteration for the MP and OMP detection algorithms, the proposed SAMP algorithm can quickly exit the iterative loop due to the non-sparse characteristics of the noise signal. According to the signal itself, the sparsity *K* can be approached quickly under the premise of unknown sparsity, thus completing the detection task, which is a problem worthy of study.

On the other hand, it can be seen that the iterative step plays an important role in the process of approaching sparsity. If the step size is smaller, the number of iterations will be larger resulting in a large amount of computation. Otherwise, the sparsity will be underestimated. In order to guarantee the detection success rate, the variable step will be adopted in future work according to the variation rule of the energy difference of the sparse vector in the adjacent phase. In the initial stage of iteration, the energy of the sparse vector decreases rapidly, thus a large step size can be adopted. When the speed of energy descent is slow, the step size should be reduced. Therefore, the time consumption of iteration will be shortened by using the idea of variable step size to close the sparsity of signal. Moreover, how to select the iterative step size is also a significant problem of further study.

Although the sparse characteristics of the target in the radar image match the theoretical model of CS, the points of a target are adjacent to each other for the linear object with a certain length. Thus, the scene from the local point of view is not sparse, which results in the sparsity of the target being reduced and then leads to affecting the detection performance. Therefore, the sparse representation of the linear object or the selection of the sparse base will be a new research direction.

## 7. Conclusions

In this paper, the application of the emerging CS theory in radar images and the geometric characteristics of the fixed targets in radar images are introduced. Then, the target detection methods for radar images based on CS is investigated. Currently, the MP and OMP detection methods demand a number of iterations based on the sparsity of the signal, and the validity of these methods are only certified based on the synthetic radar data. Then, a novel SAMP detection algorithm without the prior knowledge is proposed. The main advantage of the proposed SAMP detection algorithm is that it does not require the sparsity as the reference of the number of iterations and it realizes the detection function by expanding the support set at each stage to approach the sparsity coefficient. Based on the acquired marine radar images to verify the effectiveness of the proposed SAMP detection algorithm, the experimental results show that the SAMP target detection method can accomplish the task of signal detection without a priori information. The performance of the SAMP method is better than that of the existing MP and OMP detection algorithms. 

## Figures and Tables

**Table 1:** The parameters of X-band marine radar.

Radar Parameters	The Performance
Electromagnetic Wave Frequency	9.3 GHz
Antenna Angular Speed	22 r.p.m.
Antenna Height	25 m
Polarization	HH
Range Resolution	7.5 m
Horizontal Beam Width	0.9°
Vertical Beam Width	21°
Pulse Repetition Frequency	2000 Hz
Pulse Width	0.7°

**Table 2:** The computing time performance of various detection algorithms.

*M*	*SNR* (dB)	γ	MP		OMP		SAMP	
			$P_{d}$	*T* (s)	$P_{d}$	*T* (s)	$P_{d}$	*T* (s)
50	6	0.2	0.916	0.0702	0.974	0.0711	0.997	0.0327
100	6	0.2	1	0.0734	0.980	0.0744	1	0.0329
50	6	0.25	0.756	0.0726	0.81	0.0708	0.934	0.0328
100	6	0.25	0.702	0.0733	0.93	0.0744	0.960	0.0333
50	10	0.2	1	0.0705	1	0.0709	1	0.0327
100	10	0.2	1	0.0734	1	0.0738	1	0.0332
50	10	0.25	0.996	0.0715	0.996	0.0708	1	0.0326
100	10	0.25	0.998	0.0728	1	0.0735	1	0.0332

**Table 3:** The computing time performance of SAMP detection algorithm with various step size.

*M*	*SNR* (dB)	γ	S=2	S=4	S=6
50	6	0.25	0.0346	0.0327	0.0324
50	10	0.25	0.0346	0.0326	0.0324
100	6	0.25	0.0596	0.0333	0.0325
100	10	0.25	0.0596	0.0333	0.0325
